# Oral Function and the Oral Microbiome in the Elderly in the Kyotango Area

**DOI:** 10.3390/dj12010016

**Published:** 2024-01-18

**Authors:** Yoshiaki Yamamoto, Toshiro Yamamoto, Nao Miyamoto, Kohei Kinoshita, Satomi Nishikawa, Tetsuya Adachi, Shigeta Takizawa, Ryo Inoue, Satoaki Matoba, Narisato Kanamura

**Affiliations:** 1 Department of Dental Medicine, Graduate School of Medical Science, Kyoto Prefectural University of Medicine, Kyoto 602-8566, Japan; rutiru.yamamoto@outlook.jp (Y.Y.); n-miya@koto.kpu-m.ac.jp (N.M.); kinondent@gmail.com (K.K.); nishi-s@koto.kpu-m.ac.jp (S.N.); t-adachi@koto.kpu-m.ac.jp (T.A.); takizawa@koto.kpu-m.ac.jp (S.T.); kanamura@koto.kpu-m.ac.jp (N.K.); 2 Laboratory of Animal Science, Department of Applied Biological Sciences, Faculty of Agriculture, Setsunan University, Osaka 572-8508, Japan; ryo.inoue@setsunan.ac.jp; 3 Department of Longevity and Regional Epidemiology, Kyoto Prefectural University of Medicine, Kyoto 602-8566, Japan; matoba@koto.kpu-m.ac.jp

**Keywords:** healthy longevity, oral function, oral microbiota, oral health

## Abstract

Introduction: Prevention of tooth loss contributes to an extended life expectancy, namely longevity. Aging-related oral hypofunction, including tooth loss, markedly increases the risks of functional disorder and mortality. Dysbiosis of the oral microbiome has recently been associated with various diseases, such as liver cirrhosis, pancreatic cancer, colorectal cancer, and inflammatory bowel disease. Therefore, the relationship between the oral microbiome and systemic health has been attracting increasing attention. In the present study, we examined oral function and the oral microbiome in the elderly in a world-leading longevity area. Materials and Methods: An oral examination, chewing ability/tongue-lip motor function/saliva tests, and a metagenomic analysis with a 16S rRNA gene-targeting next-generation sequencer were conducted on 78 subjects aged ≥80 years. Twenty-six healthy individuals aged between 20 and 39 years were also investigated as controls. The data obtained were statistically analyzed. The protocol of the present study was approved by the Ethics Review Board of our university (ERB-C-885). Results: Chewing ability, tongue–lip motor function, and saliva volume were normal in elderly subjects with a current tooth number ≥20, but were significantly lower in those with a current tooth number <20. The oral microbiome in elderly subjects with a current tooth number ≥20 and young controls differed from that in elderly subjects with a current tooth number <20. Conclusion: Tooth number ≥20 in elderly subjects in the longevity area contributed to the maintenance of both oral function and the diversity of the oral microbiome.

## 1. Introduction

Healthy longevity is a universal theme for humans. According to the World Health Statistics 2020, the average life expectancy is 72.0 years worldwide and 84.2 years in Japan, which is the highest among the 194 member nations. The number of teeth is an important index of oral health. An increase in the frequency of tooth maintenance and an extension of life expectancy prevent decrease in the number of teeth [1,2,3]. In Japan, the 8020 Campaign, an educational health program run by the Ministry of Health, Labor and Welfare and the Japan Dental Association for the goal of retaining ≥20 of one’s own teeth to the age of 80 and beyond, is in operation and yielding increasingly successful results. This program is driven by the understanding that people with a greater number of original, functioning teeth have significantly better health status than those with fewer natural teeth [4]. A significant association between frailty (aging-related physical and mental weakening) in the elderly and oral frailty was found, suggesting the importance of pathological oral hypofunction, and oral frailty, which is characterized by a complex decline in oral functions, such as tongue, lip, and occlusal functions. It was evaluated using the chewing ability test, the tongue–lip motor function test, and the Saxon test [5]. According to surveys involving elderly residents, the incidence of such oral hypofunction is high [6,7,8].

Pathogenic bacteria, such as caries and periodontal pathogens, cause aspiration pneumonia [9], esophageal cancer [10], and colorectal cancer [11], and increase the risks of angina pectoris, myocardial infarction, and hemorrhagic stroke via blood flow [12]. Furthermore, aging-related oral hypofunction has been shown to markedly increase the mortality rates of cardiovascular and respiratory diseases [13]. Briefly, the maintenance of the number of teeth and oral function by control of caries and periodontal pathogens in the oral cavity may contribute to healthy longevity. A recent study suggested that caries or periodontal diseases related to these caries and periodontal pathogens lead to dysbiosis of the oral microbiome [14]. Dysbiosis of the oral microbiome has been associated with various diseases, such as colorectal cancer [15], inflammatory bowel disease [16], liver cirrhosis, and pancreatic cancer [17]; therefore, the oral microbiome has been attracting increasing attention. In the Kyotango area in Kyoto Prefecture, a world-leading longevity area, the number of individuals aged ≥100 years is 2.8-fold higher than the national average in Japan. The difference between healthy life expectancy and average life expectancy in males is approximately 1.5 years, while that in females is approximately 3 years; these values are markedly smaller than the national averages in Japan. A previous study indicated a marked difference in the intestinal flora between the Kyotango area and an urban area [18]. As demonstrated for the intestinal mucosa, the oral surface is covered by the mucosa, but the teeth erupt and penetrate the mucosa. When investigating the oral microbiome, candidate sites for samples include the tooth surface (supragingival and subgingival plaques), tongue dorsum, saliva, and oral mucosa (buccal mucosa, gingiva, and palate). In this study, we examined the oral microbiome at the tongue dorsum where the composition of bacteria is highly stable [19].

Therefore, we herein report the oral function and oral microbiome in the elderly in the Kyotango area.

## 2. Materials and Methods

Subjects of the present study consisted of 78 elderly residents in a remote area (Kyotango, a rural area bounded by sea and mountains, located on a peninsula in the north district of Kyoto Prefecture, ≥120-km from Kyoto City) and 26 healthy young residents, aged 20 to 39 years, in an urban area (Kyoto City, Kyoto Prefecture). Dentists that met the criteria standardized for examination (one dentist and one dental hygienist conducted each session, with a total of 3 dentists and 3 dental hygienists) performed face-to-face dental checkups (oral examination involving the number of teeth, state of periodontal tissue, and presence or absence of denture, chewing ability test, tongue–lip motor function test, saliva test, plaque collection) using a World Health Organization (WHO) Community Periodontal Index (CPI) probe and dental mirror according to WHO criteria under artificial lighting between November 2018 and January 2021. Plaque was collected with a dental probe on the dorsal surface of the tongue. When flaring, swelling, or pain was noted on the tongue surface, subjects were excluded from collection. Cut-off values for chewing ability/tongue–lip motor function/saliva tests were based on oral hypofunction criteria [5]. An inquiry was conducted to investigate systemic diseases, during which subjects’ height, body weight, abdominal circumference, and blood pressure were measured, and general condition was evaluated.

Subjects aged ≥80 years with ≥20 teeth were assigned to the 8020 achiever group (8020Y group), those with <20 teeth to the non-8020 achiever group (8020N group), and healthy controls to the C group. The protocol of this study was approved by the Ethics Review Board of our university (Receipt No.: ERB-C-885). The sample size of elderly subjects (volunteers aged ≥80 years) was established as approximately 80 per 2 years in reference to our previous study [18] on the intestinal flora (51 persons/year). The following check-up items were examined.

### 2.1. Evaluation of General Condition

General condition was evaluated using the Performance Status Scale prepared by the Eastern Cooperative Oncology Group (ECOG): Grade 0: Fully active, able to perform pre-disease activities without restriction; Grade 1: Restricted in physically strenuous activity but ambulatory and able to perform work of a light or sedentary nature, e.g., light house work, office work; Grade 2: Ambulatory and capable of all self-care but unable to work more than 50% of waking hours; Grade 3: Capable of only limited self-care; confined to bed or chair more than 50% of waking hours; Grade 4: Completely disabled; cannot perform any self-care; confined to bed or chair; Grade 5: Dead.

### 2.2. Chewing Ability Test

Each subject was instructed to chew a gummy jelly on the main masticatory side for 20 s, rinse the mouth with water, and spit it into a cup with a mesh filter. The filtrate was collected with a brush the tip of which was attached to glucosensor GS-II (GC, Tokyo, Japan). The measurement value displayed after 6 s was adopted as glucose elution. A value <100 mg/dL was considered to reflect reduction in chewing ability.

### 2.3. Tongue–Lip Motor Function Test

To evaluate lip or tongue movement (pa/lip motor function, ta/anterior tongue motor function, and ka/posterior tongue motor function), the number of repetitions of these syllables during a period of 5 s was measured using an oral function-measuring device (Kenko-kun Handy, Takei Scientific Instruments Co., Ltd., Niigata, Japan). The number per second was calculated. Subjects with a value <6 for one three syllables, pa, ta, and ka, were considered to have tongue–lip motor hypofunction.

### 2.4. Saliva Test

The Saxon test was used to quantitatively assess saliva. Before this test, the weight of gauze (Sterase^®^, Hakujuji Co., Ltd., Tokyo, Japan) was measured using an electron balance (BL-320S^®^, Shimadzu Corporation, Kyoto, Japan). After a chewing-like movement for 2 min, the volume of saliva absorbed by gauze was calculated by subtracting the above weight. Subjects with a 2 min saliva secretion of ≤2 g were considered to have dry mouth.

### 2.5. Plaque 16S rDNA Analysis

Dental plaque was collected, suspended in 10 µL of purified water, and stored at −20 °C until analyzed.

DNA extraction: Dental plaque was suspended in 780 µL of lysis buffer, placed in a MORA bead tube (AMR Inc., Gifu Prefecture, Japan), and mechanically crushed at a maximum rate for 3 min using an MM-400 device (Verder Scientific Co., Ltd., Tokyo, Japan). The supernatant was boiled for 5 min and automatically purified with Genefind V2 (Beckman Coulter Inc., Brea, CA, USA). DNA was eluted with 80 µL of sterile water.

Amplification of 16S rDNA V3-V4 regions (1st PCR): The V3-V4 regions of 16S rRNA were amplified using the 341F/806R primer pair and KAPA HiFi HotStart (Kapa Biosystems Ltd., London, UK). Thirty cycles were conducted under the following conditions: 95 °C (3 min) and 95 °C (30 s)/55 °C (30 s)/72 °C (30 s) followed by amplification at 72 °C (5 min). After confirming amplification, samples were purified with AmpureXP (Beckman Coulter Inc., CA, USA) and the primer was removed.

Adapter/index sequencing (2nd PCR): Necessary adapter and index sequences were provided using a primer containing an 8-base index sequence. Eight cycles were conducted under the following conditions: 95 °C (3 min) and 95 °C (30 s)/55 °C (30 s)/72 °C (30 s) followed by amplification at 72 °C (5 min). After purification with AmpureXP, electrophoresis was conducted, and samples were quantified using a Qubit fluorometer (Thermo Fisher Scientific, Waltham, MA, USA). Sample concentrations were adjusted to similar values.

Next-generation sequence: The qMiSeq method was employed. Each sample was mixed at an equivalent volume in reference to the number of reads. After adjusting the library concentration to 4 nM, the sample was degenerated with 0.2 N NaOH, and its concentration was adjusted to 10 pM using HT1 buffer. After the addition of a 5% 10 pM PhiX library solution, a next-generation sequence analysis with a MiSeq system (Illumina, Inc., San Diego, CA, USA) was performed. Results on the reads obtained were as follows: cluster density: 753 K/mm^2^, actual PhiX value: 3.52%, PassFilter: 93.42%, and Quality Score 30: 78.2% (average).

### 2.6. Sequence Data Processing

Quantitative Insights Into Microbial Ecology (QIIME) 2 (ver. 2023.2) was used to process sequence data. To denoise the data, the DADA2 plugin was used with left and right trimming lengths set at 17 and 19, respectively. The truncation length was set to 250 for both reads. The Sklearn classifier algorithm identified taxonomic assignment against the SILVA 138 database (99% Operational Taxonomic Units (OTUs) full-length sequences; available from https://docs.qiime2.org/, accessed on 27 December 2023). Singletons and ASVs assigned to mitochondria and chloroplasts were removed using the “feature-table filter-features” and “taxa filter-table” commands in QIIME2. SATé-enabled phylogenetic placement (SEPP) was used to generate the phylogenetic tree. Alpha and beta diversity metrics were calculated using the command “diversity core-metrics-phylogenetic” of QIIME2 and setting the sampling depth to 5000 reads. The Chao1 index required separate calculation using the command “diversity alpha” of QIIME2 as the “diversity core-metrics-phylogenetic” command does not calculate this index.

### 2.7. Statistical Analysis and Data Visualization

Oral function values are expressed as means ± standard deviations, and they were obtained and analyzed using GraphPad Prism 10. To compare values, one-way ANOVA and Bonferroni 120 correction with the alpha-type error set at <0.05 were used.

Alpha diversity and top 10 genus and species abundance were compared using the Wilcoxon rank sum test then visualized using R with the “microbiomeutilities” and “ggpubr” packages.

The top 20 genus abundance was visualized using R and the “MicrobiotaProcess” package. Differing taxa among groups were detected using Linear discriminant analysis Effect Size (LEfSe) analysis with a threshold of 3.0 using the “MicrobiotaProcess” package of R. Throughout the analysis, the R package “phyloseq” was used.

## 3. Results

### 3.1. Elderly Subjects

The 8020Y group consisted of 26 subjects, while the 8020N group consisted of 52. There were 6 denture-wearing subjects in the former and 50 in the latter. In the former, none had any mobile teeth. Each subject was graded zero on the ECOG PS. The details for each group are presented in Table 1.

### 3.2. Oral Function of Elderly Subjects

**Figure 1:**
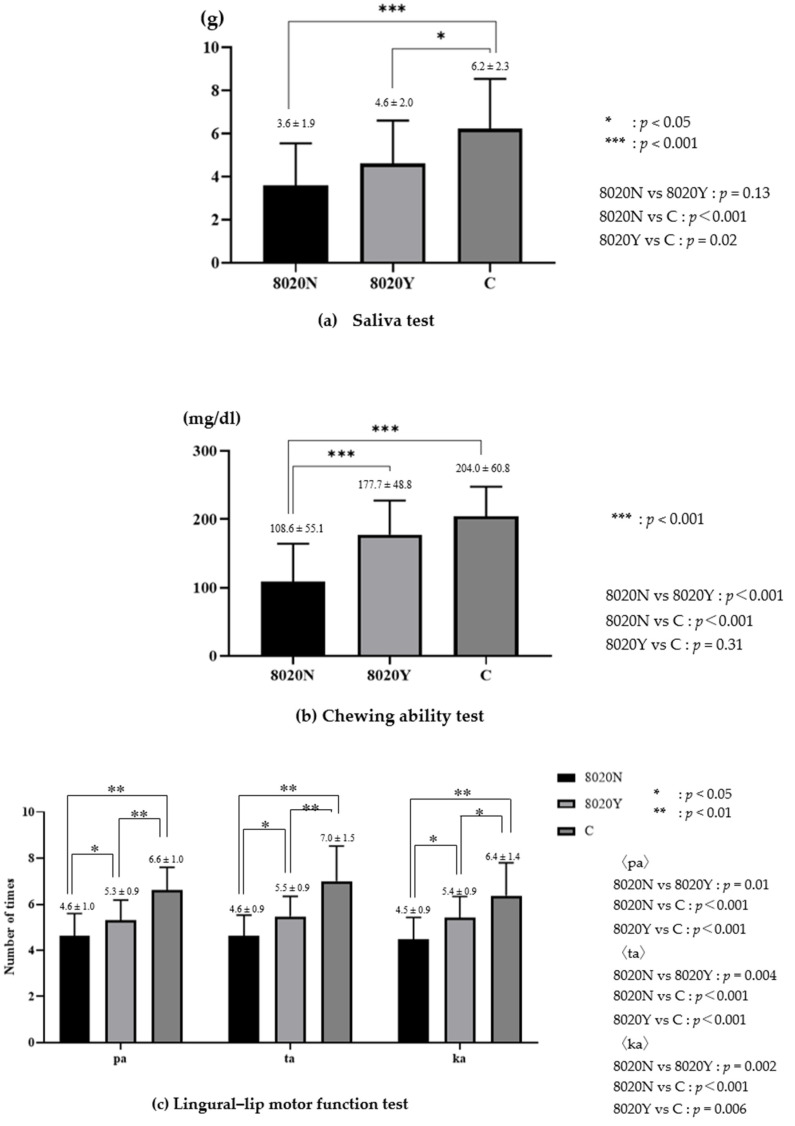
Oral function in 8020Y, 8020N, and control groups.

Chewing ability, tongue–lip motor function, and saliva volume were normal in the 8020Y group, but were significantly lower in the 8020N group (Figure 1).

### 3.3. Saliva Volume and Diversity of the Oral Microbiome

**Figure 2:**
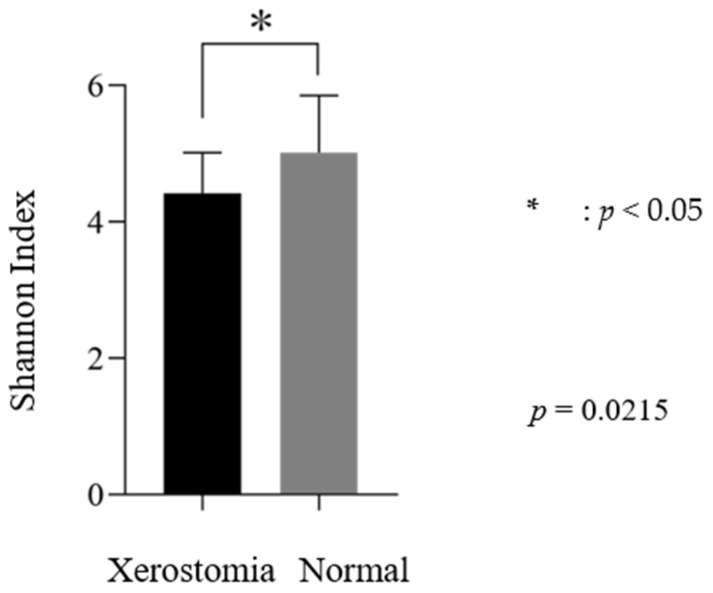
Saliva volume and diversity of the oral microbiome.

Regarding the relationship between dry mouth related to decreases in saliva volume and the diversity of the oral microbiome, diversity was significantly higher in the normal group than in the dry mouth group (Figure 2).

### 3.4. Diversity of the Oral Microbiome

**Figure 3:**
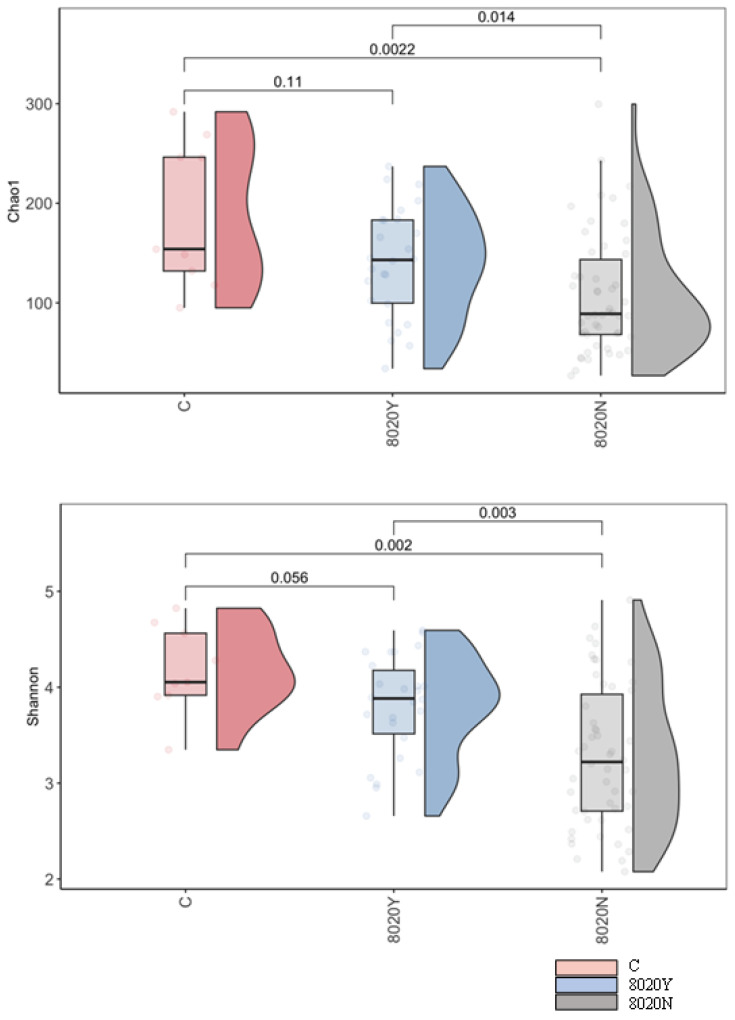
Diversity of the oral microbiome among groups.

**Figure 4:**
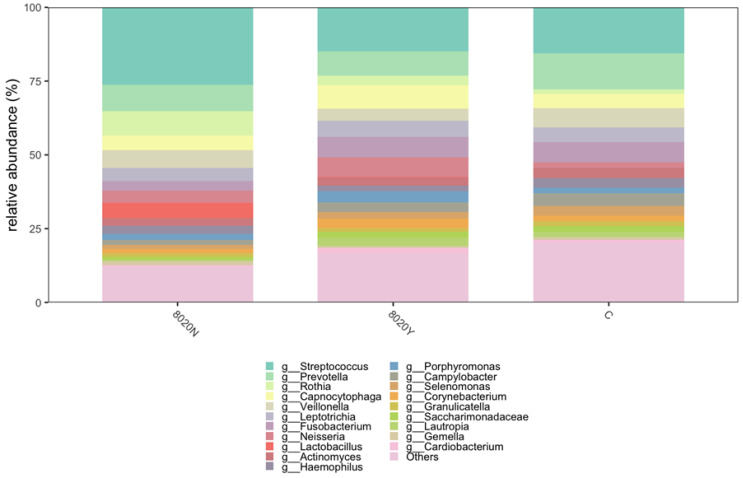
Relative abundance of the oral microbiome among groups.

**Figure 5:**
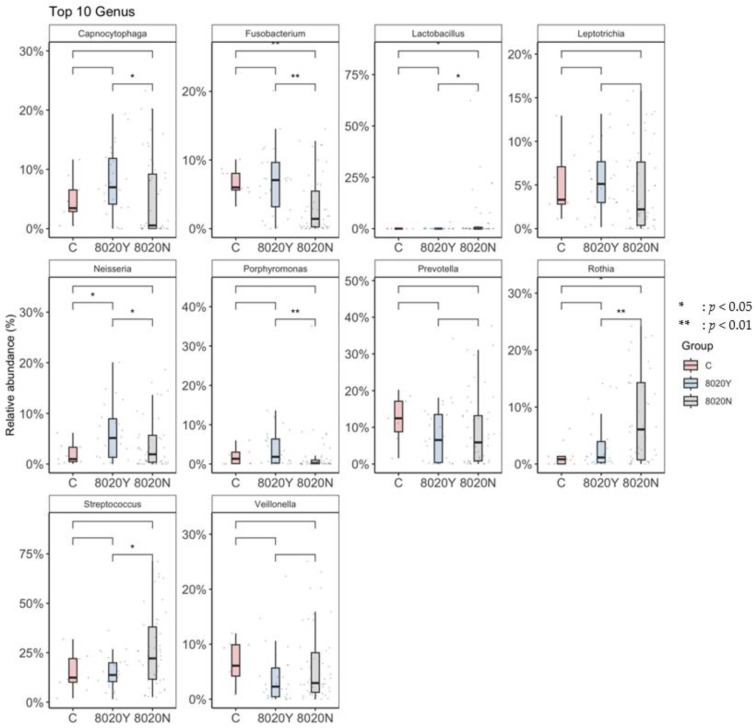
Comparison of the oral microbiome among groups (genus).

**Figure 6:**
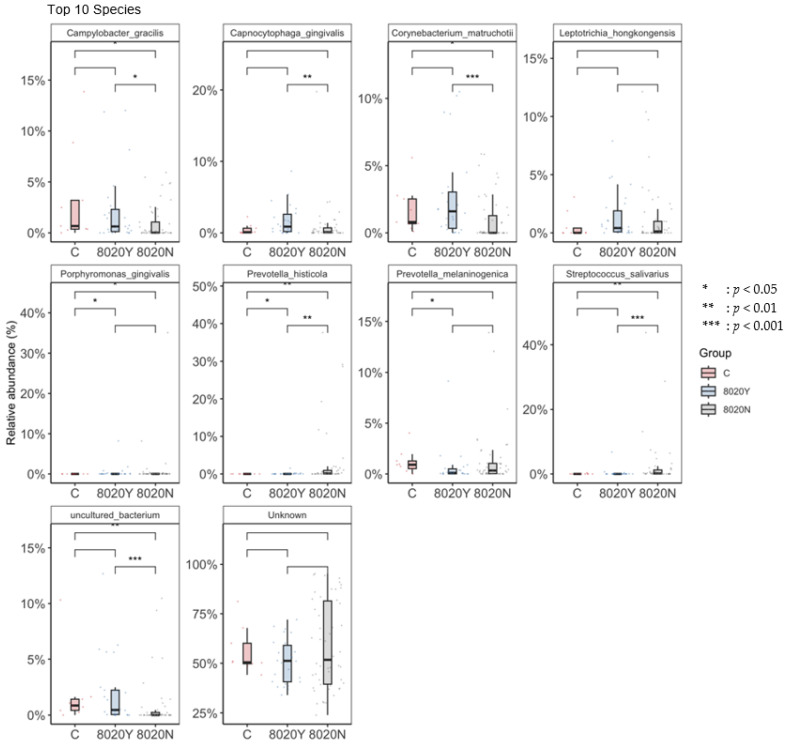
Comparison of the oral microbiome among the groups (species).

**Figure 7:**
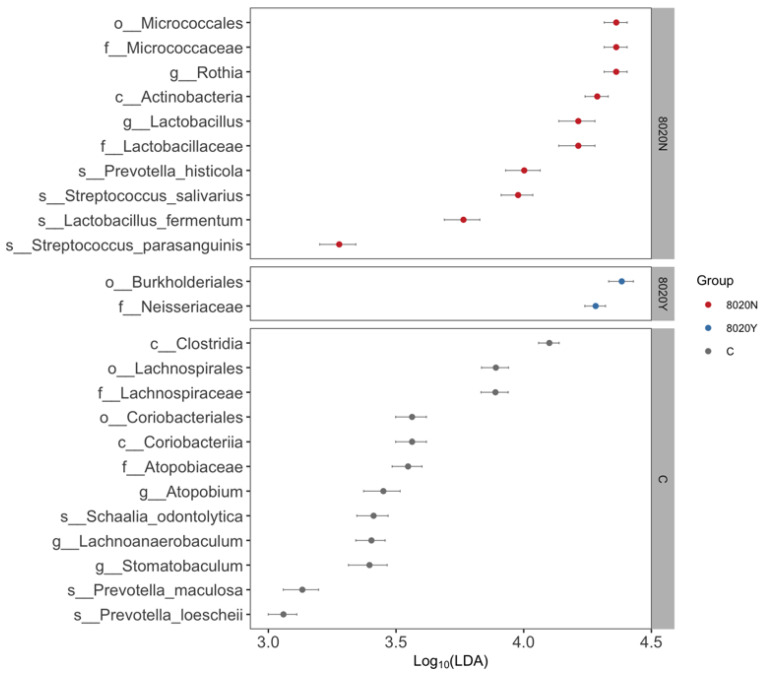
Comparison of microorganisms among groups using LEfSE.

At α-diversity analysis, significant difference was observed between the 8020Y and 8020N groups (Figure 3). Regarding taxonomic compositions, the detection rates of *Streptococcus* and *Lactobacillus* in the oral microbiome were significantly higher in the 8020N group than in the 8020Y group (Figure 4). Concerning the genus level, the detection rate of *Streptococcus* was significantly higher in the 8020N group than in the 8020Y group. Furthermore, the detection rates of periodontal pathogens, such as *Fusobacterium*, *Porphyromonas*, and *Capnocytophaga*, were significantly higher in the 8020Y group than in the 8020N group (Figure 5). Regarding the species level, the detection rate of *Porphyromonas gingivalis*, as a periodontal pathogen, was significantly higher in both the 8020N and 8020Y groups than in the C group. In addition, the detection rates of other periodontal pathogens, *Campylobacter gracilis* and *Capnocytophaga gingivalis*, were significantly higher in the 8020Y group than in the 8020N group (Figure 6). LEfSe showed that *Prevotella histicola* was abundant in the 8020N group, while other *Prevotella*, such as *P. masculosa* and *P. loescheii*, were abundant in the C group (Figure 7).

## 4. Discussion

The elderly subjects with a current tooth number <20 had significantly lower chewing ability and tongue–lip motor function than those with a current tooth number ≥20. A decrease in the number of teeth resulted in a decrease in masticatory ability [20]. In individuals with a current tooth number <20, the occlusal support region affects equilibrium, and the risk of falling in these elderly individuals not using dentures was shown to be 2.5-fold higher than in those with ≥20 teeth [21]. Furthermore, a decrease in the number of teeth or a reduction in chewing ability often leads to malnutrition [22,23]. Since reduction in oral function is associated with cognitive function [24] and social withdrawal [25], it is important for the elderly to maintain oral function.

In the present study, saliva volume was significantly lower in elderly subjects with <20 teeth than in those with ≥20 teeth. A decrease in saliva volume has been associated with drug adverse reactions, diabetes mellitus, kidney disease, Sjögren’s syndrome, radiotherapy, central/peripheral neuropathy, mental stress, aging, mouth breathing, and muscle weakness. Reduction in chewing ability has been shown to cause dry mouth [26]. In this study, tongue–lip motor hypofunction was noted, and perioral muscle weakness was suspected as an etiological factor for dry mouth. In addition to tooth surface, the oral microbiome forms on the surfaces of dental materials [27,28]. However, there is still insufficient evidence regarding its effects [29]. Based on the present results, dry mouth affected the diversity of the oral microbiome. Therefore, maintaining the current number of teeth and improving the oral hygiene environment are considered to be beneficial for oral function and the prevention of bacterial infections, such as periodontal disease. The results obtained herein suggest that a tooth number ≥20 maintained at 80 years of age is an important factor for maintaining/acquiring oral function. We note that the validity of these cutoff values was examined. This is because the decline in oral function in the elderly varies among individuals (effects of age, sex, lifestyle, medications). Therefore, when evaluating oral function, it is important to consider age and sex rather than strictly following uniform reference values [30,31].

We also compared the oral microbiome among groups. The diversity of the oral microbiome was less in the 8020N group than in the other groups. The detection rates of *Lactobacillus* and *Streptococcus* were significantly higher in the 8020N group than in the 8020Y group. *Lactobacillus* may act with *Streptococcus*, becoming highly pathogenic and causing tissue injury [32]. A previous study indicated that an increase in the salivary *Lactobacillus* count was associated with more tooth defects [33], and this was considered to be a contributing factor to the decrease in the number of teeth in this study.

In addition, the formation of denture biofilms has been associated with *Streptococcus* and *Candida* [34]. The majority of patients (50/52 patients) in the 8020N group used dentures. Saliva volume was significantly lower in the 8020N group than in the 8020Y group. A reduced saliva volume has been identified as a risk factor for *Candida* colonization [35], which may also promote the formation of denture biofilms. Since we did not examine *Candida* in each group in the present work, this issue warrants further study.

In the 8020Y group, the genus level detection rate of *Porphynomonas gingivalis*, a periodontal pathogen, was significantly higher than in the 8020N group. Additionally, in the 8020Y group, the detection rates of other periodontal pathogens, such as *Campylobacter gracilis* and *Capnocytophaga gingivalis*, at the species level were significantly higher than in the 8020N group. Pathogenic microorganisms existed at a specific rate even under an oral environment in which the number of teeth was maintained with longevity. Butyric acid produced by *P. gingivalis* has been shown to promote the progression of periodontal disease [36], whereas that produced by the intestinal flora attenuated inflammatory disease [37]. In addition, the frequency of *Prevotella* isolation was the highest among periodontal pathogenic bacteria. However, the effects of *P. masculosa*, *P. loescheii*, and *P. histicola*, which were detected in the present study, on periodontal tissue remain unclear. When carbohydrate or dietary fiber intake is high, *Prevotella* become more abundant in the intestinal flora [38]. One limitation to this study is the lack of evaluation of the relationship between the oral microbiome and intestinal flora, which is a necessary focus for future work.

An increase in the diversity of the oral microbiome is a characteristic of periodontitis [39,40]. In the present study, a relationship was not observed between the community periodontal index and the diversity of the oral microbiome as a clinical finding. The primary limitation of this study is its geographical scope, which was confined to a restricted region, specifically the rural and urban areas within Kyoto Prefecture, Japan. Therefore, these results reflect only a small population. Also, the limited sample size restricts the present study’s findings. For future studies, a larger sample number is necessary. An additional factor to be accounted for is potential variation in the oral microbiome among various oral biogeographic positions (buccal mucosa, subgingival and supragingival plaque, tongue surface).

Bioactivity, that is, quorum sensing, plays an important role in the formation of biofilms, which consist of aggregates formed by microorganisms on a solid phase surface [41]. The diversity of the oral microbiome may affect quorum sensing-mediated biofilm formation. In addition, loss of oral microbiome diversity decreases symbiotic microorganisms, increases pathogenic microorganisms, and leads to the onset of dental caries or periodontal disease. It has also been implicated in the development of atherosclerosis, Alzheimer’s disease, diabetes mellitus, autoimmune disease, and cancer [42]. Therefore, for oral health, it may be important to secure the diversity of the oral microbiome. Detailed examinations will be conducted in the future.

## 5. Conclusions

Among elderly subjects aged ≥80 years in a longevity area, favorable oral function (chewing ability, tongue–lip motor function, and saliva volume) and a diverse oral microbiome were achieved by maintaining a tooth number ≥20.

## Figures and Tables

**Table 1:** Baseline characteristics of subjects.

Number of Subjects	Kyotango 78		Kyoto 26	
Age	Male	Female	Male	Female
100–	1	0		
99–90	6	0		
89–80	34	37		
39–30			5	6
29–20			8	7
Total	41	37	13	13
Hypertension (%)	48.7 (38/78)		0	
Hypercholesterolemia (%)	33.3 (26/78)		0	
Diabetes mellitus (%)	3.8 (3/78)		0	
Cardiovascular disease (%)	6.4 (5/78)		0	
Medication (%)	24.3 (19/78)		0	

## Data Availability

The data presented in this study are available on request from the corresponding author.
